# Improvement of selenium enrichment in *Rhodotorula glutinis* X-20 through combining process optimization and selenium transport

**DOI:** 10.1080/21655979.2019.1644853

**Published:** 2019-07-26

**Authors:** Ting Wang, Xindan Lou, Genlin Zhang, Yanyan Dang

**Affiliations:** School of Chemistry and Chemical Engineering/The Key Laboratory for Green Processing of Chemical Engineering of Xinjiang Bingtuan, Shihezi University, Shihezi, China

**Keywords:** Selenium-enriched yeast, mixed carbon, enrichment, transport, R. glutinis, culture optimization

## Abstract

Selenium-enriched yeast can transform toxic inorganic selenium into absorbable organic selenium, which is of great significance for human health and pharmaceutical industry. A yeast *Rhodotorula glutinis* X-20 we obtained before has good selenium-enriched ability, but its selenium content is still low for industrial application. In this study, strategies of process optimization and transport regulation of selenium were thus employed to further improve the cell growth and selenium enrichment. Through engineering phosphate transporters from *Saccharomyces cerevisiae* into *R. glutinis* X-20, the selenium content was increased by 21.1%. Through using mixed carbon culture (20 g L^−1^, glycerol: glucose 3:7), both biomass and selenium content were finally increased to 5.3 g L^−1^ and 5349.6 µg g^−1^ (cell dry weight, DWC), which were 1.14 folds and 6.77 folds compared to their original values, respectively. Our results indicate that high selenium-enrichment ability and biomass production can be achieved through combining process optimization and regulation of selenium transport.

## Graphical Abstract

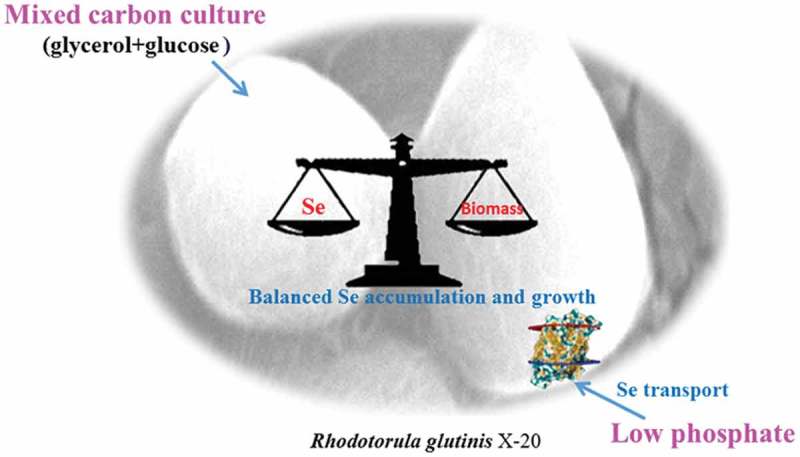

## Introduction

Selenium (Se) is a trace element essential for human health [1–3]. The World Health Organization (WHO) has reported that selenium deficiency is one of the most universal nutritional deficiencies in the world [4]. Selenium deficiency affects approximately 0.5–1 billion people around the world and is prone to causing health disorders such as cancer, Keshan disease, Kashin-Beck disease, muscular syndrome, and even death [3–5]. Selenium in the body is mainly supplied through diet [5], however, many foods are selenium deficient due to their planting environment, e.g. approximate 72% of Chinese cultivated land is selenium deficient (<0.04 mg kg^−1^), with at least 30% being severely selenium deficient. Currently, the average dietary intake of selenium by Chinese adults is only 26–32 µg per day, which is much less than the WHO-recommended intake of 40–200 µg per day [2]. Therefore, developing selenium-enriched products is of great significance to improve health standards, especially of communities whose nutrition is absent or deficient in organic selenium.

Among the various selenium-enriched products, selenium-enriched yeast has been considered a safe and effective nutrient fortifier. Previous studies have verified that the dietary supplementation of selenium-enriched yeast greatly improved health compared to those who were administered inorganic selenium products [6]. Moreover, yeast’s ability to incorporate metals has been exploited as a delivery vehicle for many minerals [7]. However, the imbalance between cell growth and selenium accumulation usually attenuates the quality of selenium-enriched yeast [8–10]. Excessive amounts of selenium prevent enzymatic oxidation of yeast macromolecules [11], and high concentrations of the selenide form of selenium can be particularly toxic to cells [12,13]. Although several approaches have been tested to solve this problem, there are still tremendous challenges. For example, treating yeast with a pulsed electric field increased selenium accumulation by 65%, but resulted in a threefold reduction in biomass, possibly due to cell toxicity or changed enzymatic activity [14]. Although supplementing selenium at different stages in the cell cycle can reduce the toxicity of higher concentrations, biomass still decreased by up to 20% [15]. Because the mechanism of coordinating selenium accumulation and yeast growth is not fully understood, developing a method that can balance these processes is extremely important.

Theoretically, yeast accumulates selenium via physicochemical and biological mechanisms, including extracellular binding by metabolites and biopolymers, binding to the specific polypeptides, and metabolism-dependent accumulation, however, the capability of yeast to enrich selenium largely depends on their endogenous characteristics [7]. The most classical and effective way to obtain a highly selenium-enriched yeast is still screening the strain from a selenium-enriched environment. In 2008, a geological survey of China discovered a large area containing selenium-enriched soil (selenium content ranging from 0.14–2.75 mg kg^−1^) on the north slope of the Tianshan Mountains in Xinjiang, providing a possible environment for selenium-enriched microorganisms [16,17]. From this area, we have identified a strain of yeast, *Rhodotorula glutinis* X-20, which shows good selenium enrichment and growth performance [18]. The objective of the present study is to optimize fermentation conditions and to balance cell growth and selenium enrichment. Basic culture conditions are established and process optimization is performed. The selenium-enrichment capacity of *R. glutinis* X-20 is greatly improved by regulating the phosphate transport system, and selenium enrichment and cell growth is also balanced by using a mixed carbon source in culture. This study provides a foundation for the application of *R. glutinis* X-20.

## Materials and methods

### Strain and medium

*R. glutinis* X-20 colonies were inoculated in yeast extract peptone dextrose medium (YPD medium, containing 1% yeast extract, 2% peptone, and 2% glucose) with 30 mg·L^−1^ sodium selenite. After culturing 36 h at 30°C, their selenium-enrichment potential was determined with Hydride Generation Atomic Fluorescence Spectrometry (HG-AFS).

### Determination of selenium content in yeast

The total selenium content in yeast was determined by Hydride Generation Atomic Fluorescence Spectrometry (HG-AFS) [19–21]. One millilitre of yeast broth was centrifuged at 8,000 rpm for 10 min and washed 3 times with deionized water to remove selenium ions and selenium salt that were unbound to yeast cells. The collected cells were transferred into a Teflon crucible. 10 mL mixed acid (9 mL HNO_3_ and 1 mL perchloric acid) and few glass beads were added sequentially. The cells were digested at 200°C using an electric heating plate until the solution volume was reduced to 2 mL. The digested solution was cooled to room temperature, and 5 mL hydrochloric acid (6 mol L^−1^) was added to reduce selenium (VI) into selenium (IV) before hydride generation. The digested solution was then transferred into volumetric flasks and diluted with ultrapure water to 50 mL. A blank control solution was prepared using the same method. The digested product was used for total selenium determination according to the HG-AFS conditions described in Table 1.

**Table 1.** HG-AFS conditions.

High negative voltage of PMT (V)	340
Atomizer height (mm)	8
Lamp current (mA)	100
Se-HCL, wavelength (nm)	196
Flow rate of carrier gas (argon) (mL·min^−1^)	400
Flow rate of shield gas (argon) (mL·min^−1^)	800
Flow of reducing solution (NaBH_4_) (mL·min^−1^)	4
Read method	Peak high
Read time (s)	6

For extraction and determination of inorganic selenium, cells were mixed with ultrapure water and boiled for 1 h. After centrifugation at 8,000 rpm for 15 min, the supernatant was filtrated and used to evaluate the inorganic selenium content. Organic selenium content in yeast was calculated by taking the difference between total selenium content minus inorganic selenium content [22].

### Assay of biomass

Biomass was estimated through measuring the optical density at 600 nm. The dry weight of the cell (DWC) was calculated according to the relationship between the DWC and OD_600_. To determine the DWC, 5 mL yeast culture was added into a weighed centrifuge tube, centrifuged for 5 min at 10,000 rpm and the supernatant was discarded. After three washes with sterile water, the centrifuge tube containing yeast cells was dried at 105°C until it reached a constant weight (the final weight). The DWC was obtained by subtracting the blank tube weight from the final weight. Meanwhile, the optical density at 600 nm of the cells was recorded. Each experiment was performed in triplicate. The linear relationship between DWC and OD_600_ is shown in Equation 1.

(1) $$DWC=0.2025\times OD_{600}+0.5127$$

### Plasmid construction

To study the effect of selenium transport on yeast growth and selenium enrichment, four phosphate transporters Pho84, Pho87, Pho89, and Pho90, were selected by searching the National Centre for Biotechnology Information (NCBI) database. Pho84, Pho87, Pho89, Pho90, promoter TYS1, and the ATP15 terminator were cloned via PCR from the *S. cerevisiae* CEN.PK2-1C genome. Promoter TYS1, PhoX, and terminator ATP15 were ligated by OE-PCR. After Hind III and Sal I digestion, the ligated products were inserted into the pRS42K plasmid linearized by Hind III and Sal I to form the new plasmid pRS42K-phoX. Recombinant plasmids were transformed into yeast by an improved lithium acetate transformation method. Transformants were screened through resistance screening and colony PCR.

### Quantitative PCR

Total RNA extraction, first-strand cDNA synthesis, and qPCR analysis were performed as previously described [23]. PCR conditions consisted of 40 cycles at 95°C for 5 sec, 60°C for 20 sec, and 72°C for 1 min, with a final extension at 72°C for 10 min. ACT1 was used as the reference gene, and relative transcription levels were calculated based on the level of ACT1 mRNA.

## Results and discussion

Selenium yeast is important for health and the pharmaceutical industry. Previously, yeast strain *R. glutinis* X-20, which showed good selenium enrichment and growth performance, was screened from selenium-enriched soil in Xinjiang, China. Optimal culture conditions were determined through single experimental factors [18]. To further optimize the selenium enrichment of this yeast, basic culture conditions including temperature, pH, culture time, and liquid volume were explored. Because selenite absorption depends on transporters of phosphate or monocarboxylate [7], phosphorus stimulation and a transformed phosphate transport system were employed to improve selenium enrichment. Different carbon sources can have different effects on cell growth and selenium metabolism, thus mixed carbon culture was employed to balance cell growth and selenium enrichment. After optimization, a selenium content of 5349.6 µg g-1(DWC) was obtained.

### Basic culture conditions and process optimization

#### Temperature and pH

**Figure 1.**
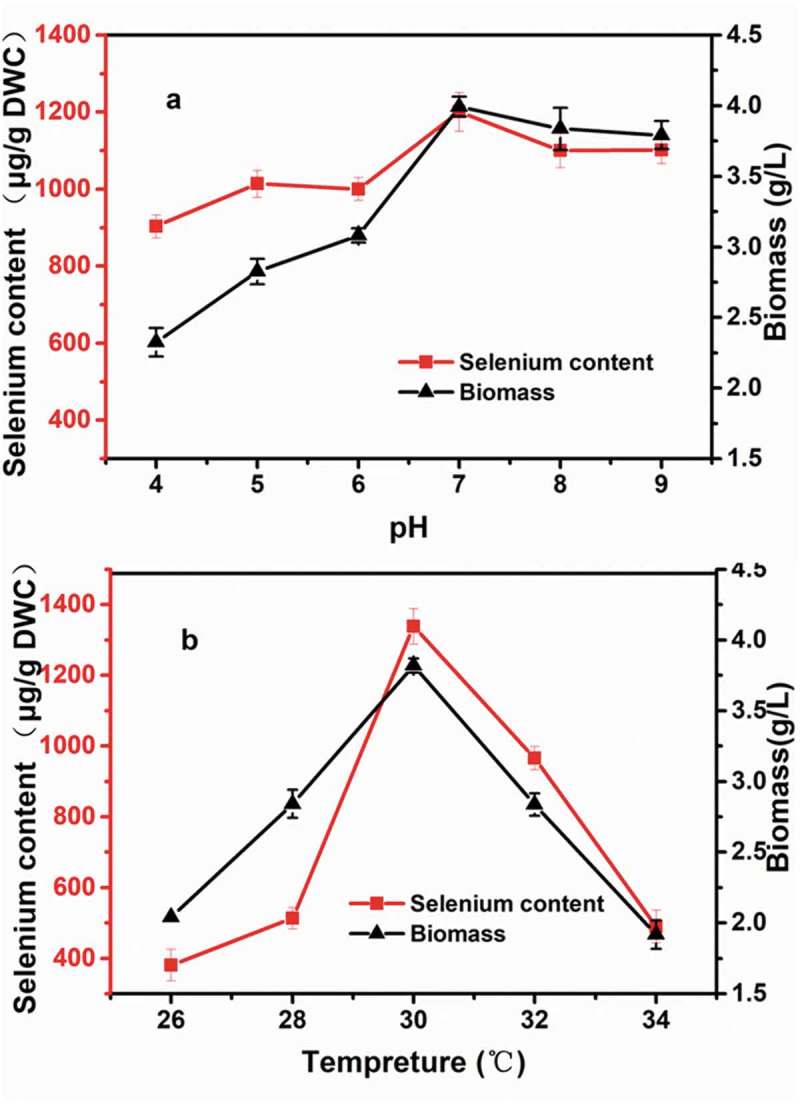
Influence of pH (a) and temperature (b) on the biomass and selenium content of *R. glutinis* X-20.

Temperature and pH are highly significant for yeast growth and selenium enrichment. Figure 1(a) shows that the biomass and selenium-enrichment potential of strain X-20 are highly pH dependent. The X-20 strain accumulated a maximum amount of selenium when grown at pH 7.0. However, earlier studies have reported that selenium accumulation in yeast was more efficient at lower pH values [24]. Yin et al. also demonstrated that the optimal pH for selenium enrichment in *S. cerevisiae* was 5.822. Using *Rhodotorula*, the optimal pH for enrichment of other ions was also between 6.0 and 7.0 [25]. Efficient selenium enrichment at pH 7.0 implies an underlying difference in mechanism of selenium accumulation in *R. glutinis* X-20, which should be further examined. The cell growth and selenium accumulation are substantially increased when the culture temperature raised from 26°C to 30°C (Figure 1(b)). Over 30°C, there is a steep decrease in both selenium content and biomass. The selenium content and DWC of *R. glutinis* X-20 at 30°C reached 1338.9 µg g^−1^ and 3.82 g L^−1^, respectively. This culture temperature is similar as the other selenium-enrichment yeasts such as *S. cerevisiae* [26,27].

#### Culture time

**Figure 2.**
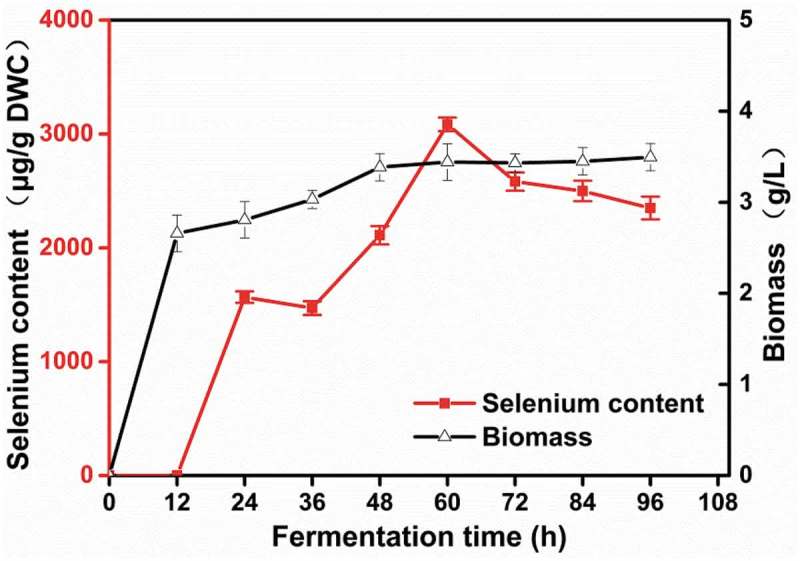
Influence of fermentation time on the biomass and selenium content of *R. glutinis* X-20.

Fermentation time not only affects selenium enrichment but also influences production cost. To explore the optimal fermentation time, *R. glutinis* X-20 was inoculated in 50 mL fresh YPD medium (with 2% glycerol as a carbon source and peptone as a nitrogen source) in ﬂasks with 1% inoculation. After 12 h culture at 30°C and 200 rpm, 50 g/mL sodium selenite was added into the ﬂasks. From 24 h to 60 h, selenium content and yeast biomass increased by 96.919% and 63.629%. With the prolonged culture time, selenium content decreased sharply while yeast biomass remained stable (Figure 2). These results suggest that selenium accumulation in *R. glutinis* X-20 is growth-related. From an economic perspective, fermentation time is a very important factor for production [28]. Shortening fermentation time can shorten the production cycle of industrial selenium-enriched yeast and increase utilization of the instrument. Thus, in follow-up experiments we decided to use 60 h as an endpoint for fermentation.

#### The volume of liquid

**Figure 3.**
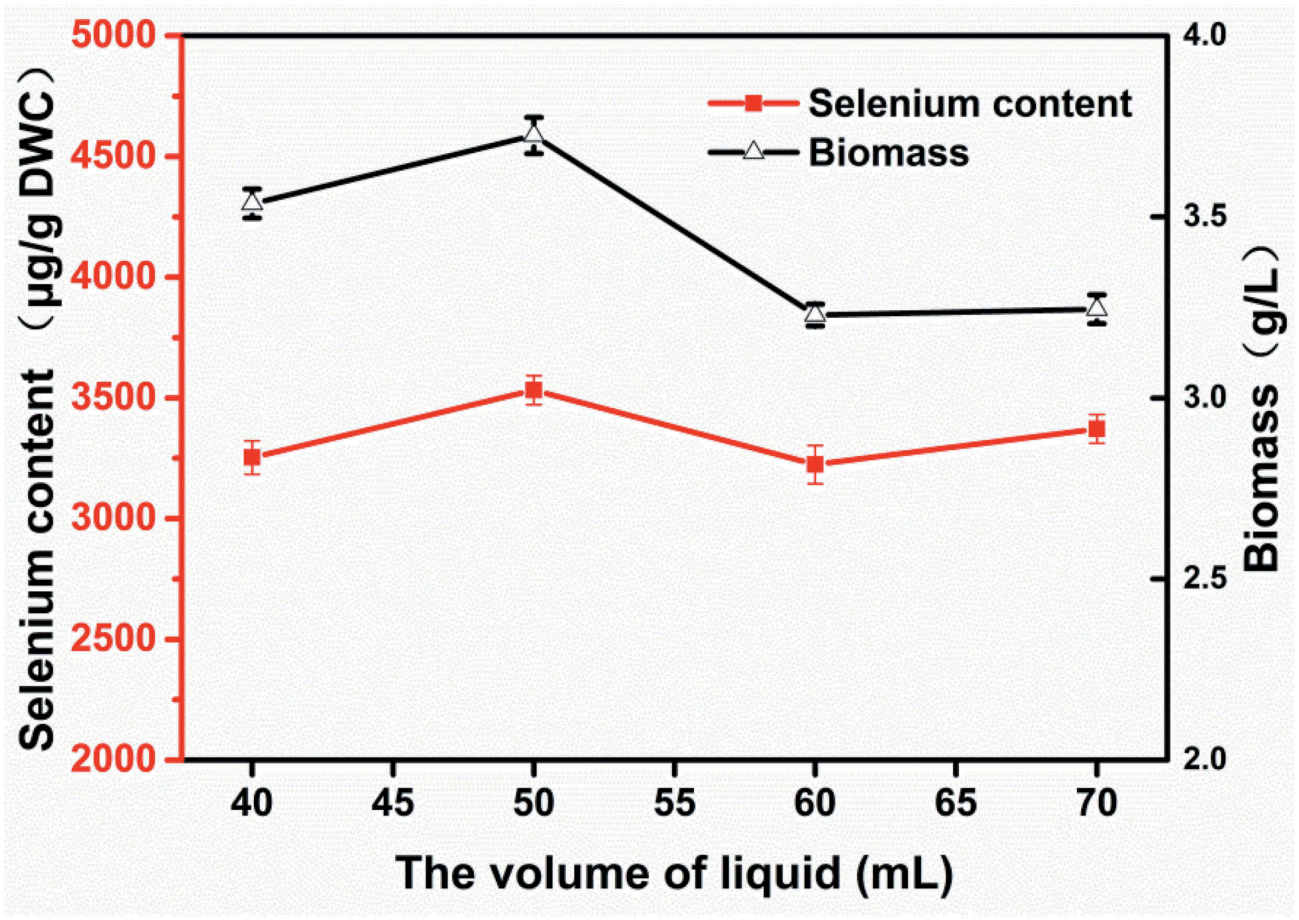
Influence of the volume of liquid on the biomass and selenium accumulation of *R. glutinis* X-20.

To explore the effect of dissolved oxygen on selenium enrichment, different volumes of YPD media were added to 250 mL shake flasks. Figure 3 shows that larger liquid volumes had a negative effect on selenium enrichment, while smaller liquid volumes were better for the growth and selenium enrichment of *R. glutinis* X-20, with 50 mL media in a 250 mL flask as the optimal volume. These results are consistent with previous work [29]. Fermentation in shaking flasks with less liquid has higher a volumetric dissolved oxygen coefficient value but increases the evaporation of the fermentation broth. Excessive oxygen is not conducive to the propagation and growth of yeast and will inhibit its selenium-enrichment capabilities [30]. Therefore, 50 mL/250 mL was used for subsequent experiments.

### Improving selenium enrichment by regulating phosphate transport system

In yeast, selenite is absorbed in a metabolism-dependent manner using transporters of phosphate or monocarboxylate [7]. In our previous work, we demonstrated that the addition of phosphorus increases the selenium content of yeast [18]. When *R. glutinis* X-20 was grown in medium containing 0.4 mM phosphate, it increased selenium enrichment to 3909 µg g^−1^ DWC. Less than 0.4 mM phosphate promoted cell growth and selenium enrichment while more than 0.4 mM phosphate inhibited it. These findings suggest that a low concentration of phosphate enhances the uptake and tolerance of selenium in yeast [31,32]. Phosphorus’ ability to regulate selenium enrichment may be related to selenium transport in yeast. According to reports, both high and low affinity phosphate transporters are supposedly involved in the transport of selenium [33]. The low affinity transport system, composed of Pho87 and Pho90, operates at higher phosphate concentrations (>1 mM), while at low concentrations, it is post-transcriptionally downregulated through vacuolar targeting by Spl2 (also a member of the PHO regulon) [34,35]; the high affinity transport system composed of Pho84 and Pho89 functions at both low and high phosphate concentrations, depending on the PHO pathway [36]. Therefore, under high phosphate conditions, Pho87 and Pho90 are the main transporters of selenite, while at low phosphate condition Pho84 and Pho89 are the main selenite transporters.

**Figure 4.**
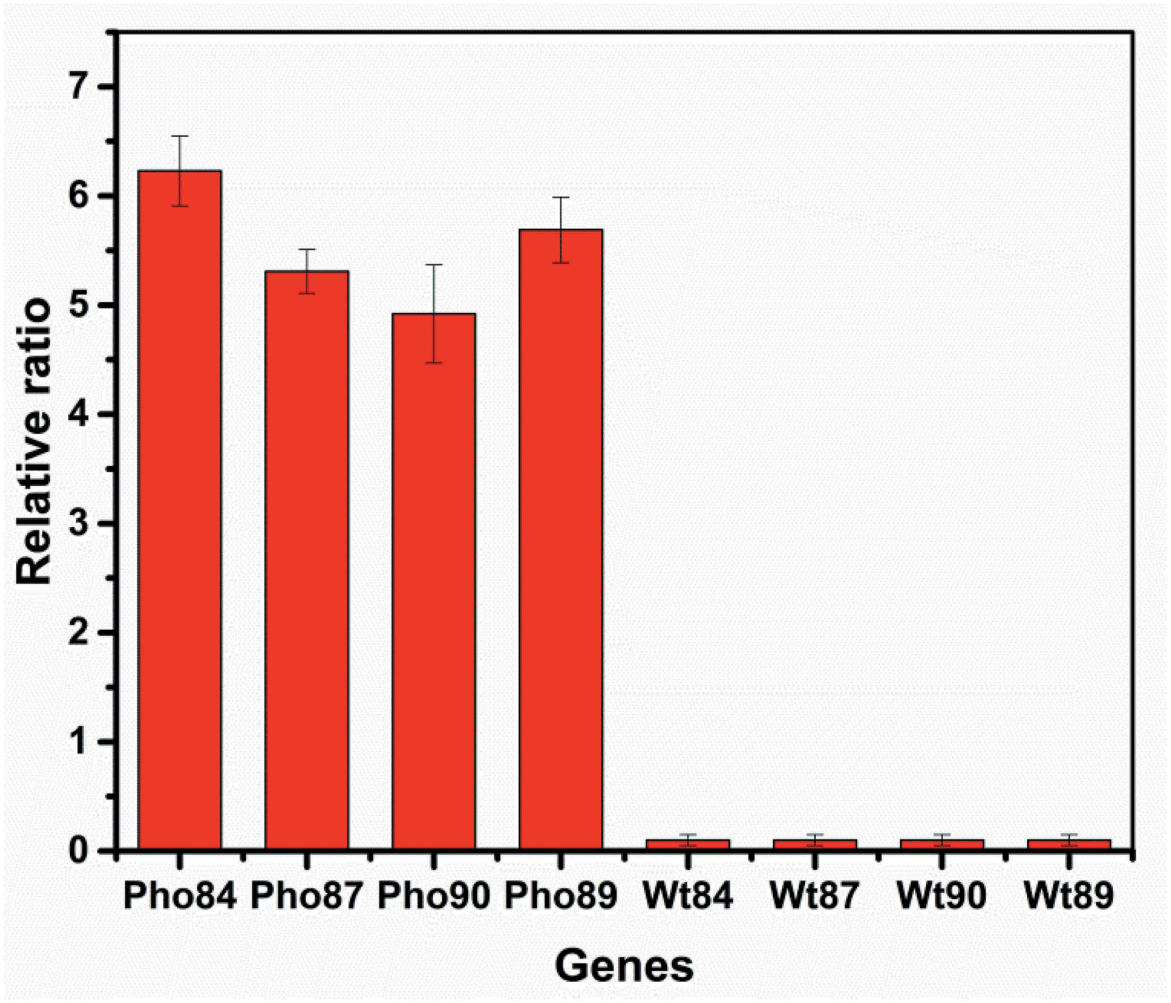
Transcriptional analysis of Pho84, Pho89, Pho87 and Pho90.

**Figure 5.**
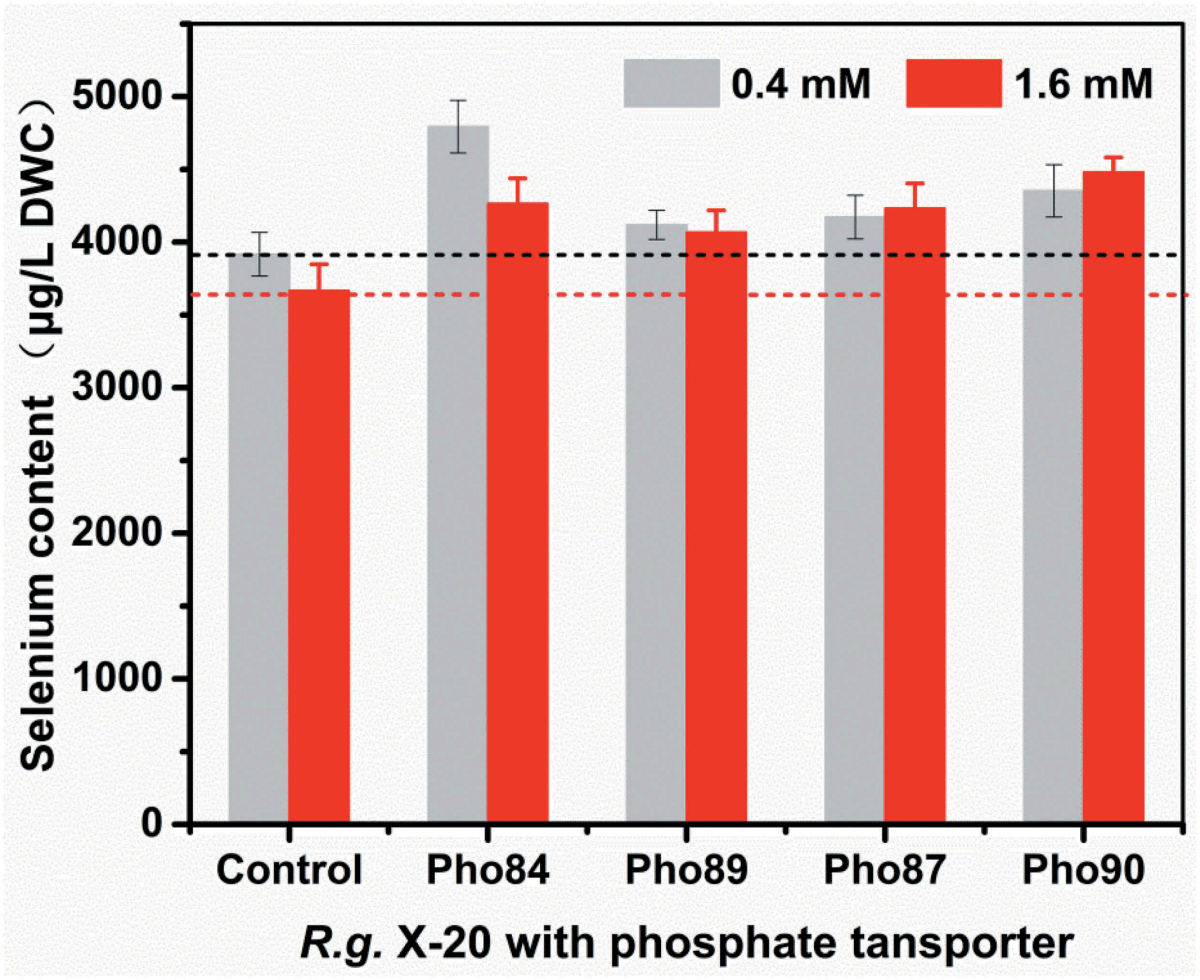
Effects of phosphorus transporter on selenium accumulation in *R. glutinis* X-20.

To investigate the relationship between phosphate transporters and selenium transport, we transformed Pho84, Pho89, Pho87, and Pho90 from *S. cerevisiae* into *R. glutinis* X-20. As shown in Figure 4, qPCR confirmed that the heterologous Pho84, Pho89, Pho87, and Pho90 are effectively expressed in *R. glutinis* X-20. Due to the absence of these genes in *R. glutinis* X-20, no transcriptional data were detected (wt84, wt87, wt90, wt89). Compared with the control, the selenium content in yeast with these transformed transporter genes increased (Figure 5). As expected, Pho84 and Pho89 promote selenium enrichment at a low phosphorus concentration (0.4 mM), whereas Pho87 and Pho90 show good effect at a high concentration (1.6 mM). The total selenium reached 4752 µg·g^−1^ DWC when Pho84 was expressed in *R. glutinis* X-20. Therefore, selenium enrichment in yeast can be effectively improved by phosphorus stimulation or by regulating the phosphate transport system.

### Balancing cell growth and selenium enrichment by mixed carbon culture

**Figure 6.**
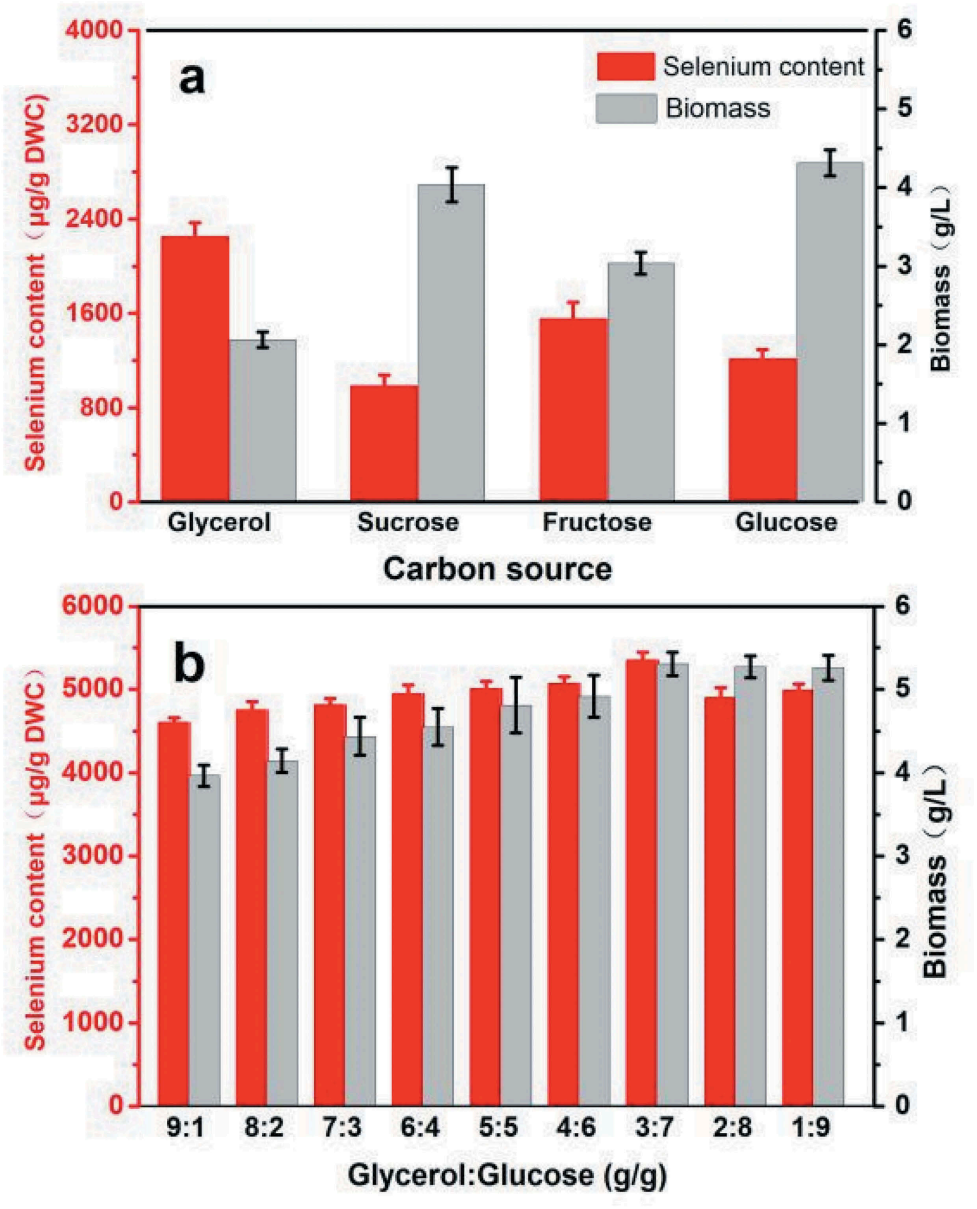
The effect of mixed carbon source on the biomass and selenium accumulation of *R. glutinis* X-20.

Carbon source identity contributes to the proliferation of yeast cell and can affect selenium accumulation and cell growth. As illustrated in Figure 6(a), *R. glutinis* X-20 achieves the highest biomass when cultivated using glucose as the sole carbon source, whereas its selenium content shows maximum when glycerol is the sole carbon source, indicating an imbalance between selenium accumulation and cell growth. Therefore, an alternative culture using a mixed carbon source was further designed. Figure 6(b) shows the selenium enrichment and biomass production of *R. glutinis* X-20 after 60 h using mixed glycerol and glucose. Yeast growth increases with increasing of glucose content. When the ratio of glycerol to glucose was 3:7, both biomass and selenium content achieved a maximum, 5.30 g L^−1^ and 5349.6 µg g^−1^ DWC, which were 1.14 folds and 6.77 folds compared to their original values, respectively. Ethanol and hydrogen peroxide by-products from glycerol could strengthen the resistance of cells to the external environment and regulate the balance of cell osmotic pressure produced by high concentrations of selenium and their oxidation-reduction potential, improving selenium accumulation [37,38]. The comparative analysis showed that the selenium content of *R. glutinis* X-20 is 2–5 times higher than most reported yeasts, except for mixed strains (Table 2). This evidence indicates that the imbalance between growth and selenium enrichment of *R. glutinis* X-20 could be resolved using the simple culture method of a mixed carbon source.

**Table 2.** Selenium-enriched performance in various yeasts.

Strain	Selenium content (µg/g)	Methods	Reference
*R. glutinis* X-20	5349.6	Regulating selenium transport and using mixed carbon culture	This study
*S. cerevisiae* YR166	2608.7	60 Co ץ-ray irradiation methods	[27]
*S. cerevisiae*	2452	Mutagenesis	[39]
*S. cerevisiae*	1400	Optimization of cultivation conditions	[15]
*S. cerevisiae* ATCC MYA-2200 +* C. utilis* ATCC 9950	5470	Adding sodium selenite salts of different concentrations	[10]
*C. utilis* CCTCC M 209298	1080	Acid stress	[40]

## Conclusion

Selenium-enriched yeast has been considered a safe and more effective nutrient fortifier, but an imbalance between yeast cell growth and selenium accumulation usually leads to lower product quality. Our study demonstrates that supplementation with phosphate could trigger the uptake of selenite by phosphorus transporters. The culture conditions of *R. glutinis* X-20 were optimized in terms of pH, culture time, and dissolved oxygen content. Using a simple mixed carbon source (glycerol and glucose in a ratio of 3:7) culture, selenium enrichment and cell growth can be efficiently balanced, finally obtaining 5349.6 µg·g^−1^ DWC selenium content. These methods can be employed at large scale for selenium enrichment of yeast for health and pharmaceutical settings.

## Article Highlights

- Explore culture method for balancing cell growth and selenium-enriched capability.
- Mixed carbon source and low phosphate to regulate the selenite transportation.
- The selenium content reach the maximum is 5349.6 µg/g in *Rhodotorula glutinis* X-20.
